# Carbon nanodot precursors enable ultrahigh-loading copper single-atom catalysts for oxygen reduction reaction

**DOI:** 10.1039/d6ra01912a

**Published:** 2026-07-06

**Authors:** Prakhar Sharma, Ayanthi Thisera, Matthew G. Boebinger, Jenna K. Rector, Jason M. Unrine, Beth S. Guiton, Doo Young Kim

**Affiliations:** a Department of Chemistry, University of Kentucky Lexington Kentucky 40506 USA dooyoung.kim@uky.edu; b Center for Nanophase Materials Sciences, Oak Ridge National Laboratory Oak Ridge Tennessee 37831 USA; c Department of Plant and Soil Sciences, University of Kentucky Lexington Kentucky 40506 USA

## Abstract

Single-atom catalysts (SACs) have attracted significant attention in electrocatalysis due to their high metal utilization and tunable electronic structures; however, their practical implementation is often limited by poor atomic dispersion, low metal loadings (typically < 1 wt%), and insufficient anchoring of single metal atoms on catalyst supports. Herein, we report a simple and versatile strategy to synthesize copper single-atom catalysts (Cu-SACs) with ultrahigh metal loading using citric-acid-derived carbon nanodots as metal-capturing precursors. Through hydrothermal treatment followed by pyrolysis in the presence of urea, atomically dispersed copper atoms coordinated with nitrogen are incorporated into a carbon framework, achieving a Cu loading of up to 20 wt% while maintaining atomic dispersion. Notably, a significant fraction of the copper single atoms exists as Cu^+^–N_2_ coordination, which provides under-coordinated and catalytically active sites. The resulting Cu-SAC exhibits promising oxygen reduction reaction (ORR) activity in alkaline media, delivering a limiting current density comparable to that of commercial 20 wt% Pt/C with a predominant four-electron reduction pathway. Despite this high intrinsic activity, durability tests reveal gradual performance degradation under prolonged electrochemical operation, which is attributed to demetallation and aggregation of copper single atoms into metallic nanoparticles. These results demonstrate the potential of carbon nanodot-based platforms for synthesizing high-loading SACs, while underscoring the critical need to simultaneously control carbon porosity and metal–nitrogen coordination to enhance active-site accessibility and durability.

## Abstract 2

Synthesis of Cu single atom catalysts (Cu-SAC) with citric-acid-derived carbon nanodots (CNDs).

## Graphical abstract

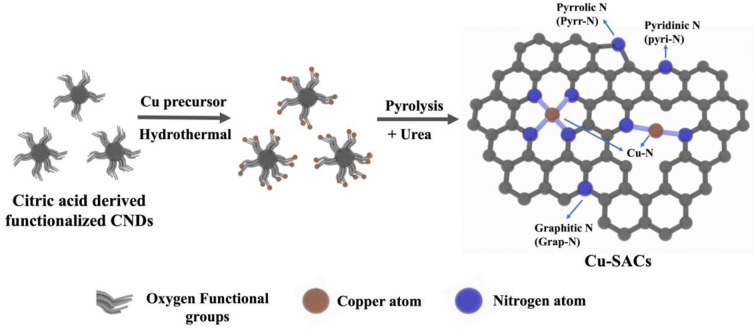

## Introduction

Single-atom catalysts (SACs) have emerged as a compelling class of functional materials due to their ability to maximize metal utilization while offering well-defined and tunable active sites for catalysis.^1–4^ In particular, carbon-supported metal–nitrogen coordination motifs (M–N–C) have attracted extensive interest for electrochemical reactions, as isolated metal centers can exhibit distinct electronic structures compared to metal nanoparticles.^5–8^ Common approaches for synthesizing M–N–C catalysts include impregnation–pyrolysis,^9–12^ metal–organic framework (MOF)-derived strategies,^13–17^ and atomic layer deposition (ALD).^18–20^ While these methods can generate metal–nitrogen coordination structures, achieving high metal loading with uniform atomic dispersion remains challenging due to aggregation during high-temperature treatment or limited deposition per cycle.

Beyond synthesis strategies, stabilizing high-loading single-atom catalysts while preserving accessible active sites represents a fundamental materials challenge. Increasing metal content requires a sufficient density of anchoring sites and robust metal–nitrogen coordination to prevent thermodynamically favored aggregation into nanoparticles. Simultaneously, the carbon framework must provide appropriate porosity and structural stability to maintain active-site accessibility under electrochemical conditions. Achieving this balance between metal loading, coordination stability, and structural integrity remains a critical barrier to the practical development of SAC systems.

Here, we report a simple and versatile strategy for synthesizing copper single-atom catalysts (Cu-SACs) using citric-acid-derived carbon nanodots (CNDs) as atom-capturing and nitrogen-rich precursors. CNDs provide a high density of oxygen-containing functional groups for molecular-level metal capture,^21,22^ while subsequent pyrolysis in the presence of urea introduces nitrogen dopants and converts the CNDs into a conductive carbon framework. This process stabilizes isolated Cu atoms through Cu–N coordination and enables atomically dispersed copper at loadings as high as 20 wt% without the need for complex templating or deposition techniques.

The resulting Cu-SACs exhibit uniform atomic dispersion, high nitrogen content, and well-defined Cu–N coordination environments, as confirmed by aberration-corrected STEM, X-ray photoelectron spectroscopy, and Auger spectroscopy. Electrochemical oxygen reduction reaction (ORR) is employed as a model reaction to probe the accessibility and activity of the Cu single-atom sites, with the optimized Cu-SAC displaying ORR activity comparable to commercial Pt/C in alkaline media. Prolonged electrochemical cycling reveals gradual demetallation and nanoparticle formation, providing mechanistic insight into the stability limitations of Cu–N–C single-atom motifs and underscoring the critical need for precise control over metal–ligand coordination and carbon microstructure.

## Experimental

### Materials

Citric acid (ACS reagent, 99.5%) was purchased from Sigma-Aldrich. Copper(ii) sulfate, anhydrous (99.5%) and iron(iii) nitrate nonahydrate (ACS, 98.4%) were obtained from J. T. Baker. Urea (ACS) was bought from MP Biomedicals, LLC. All reagents were used as it is without any further purification. Commercial 20 wt% platinum carbon on Vulcan was purchased from Premetek Co. and was used for ORR comparative purposes.

### Synthesis of Cu-SACs

**Fig. 1:**
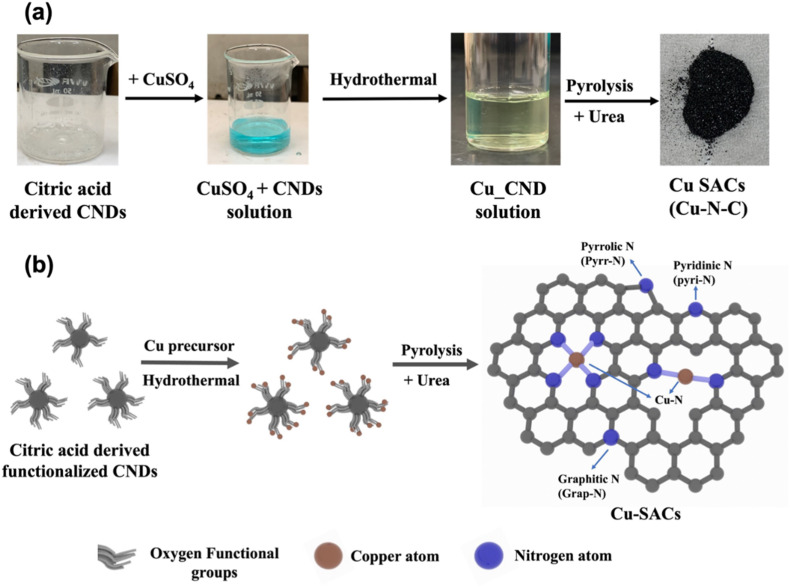
(a) Synthesis procedure to obtain Cu-SACs and (b) synthesis scheme.

The overall synthesis procedure for SACs is illustrated in Fig. 1. CNDs provide a high density of oxygen-containing functional groups for copper capture. A subsequent pyrolysis in the presence of urea introduces nitrogen dopants and converts the CNDs into a conductive carbon framework while Cu atoms are stabilized by Cu–N coordination (Fig. 1). In a typical synthesis, *X* mg (*X* = 500, 250, or 150 mg) of citric acid (CA) was placed in a 50 mL beaker and heated on a hotplate at 160–180 °C until fully melted. During heating, gas evolution occurred due to dehydration and decarboxylation of CA, accompanied by a color change of the melt from white to pale yellow. Subsequently, 10 mL of an aqueous CuSO_4_ solution (10 mg mL^−1^) was added to the molten CA, yielding a homogeneous blue solution.

The resulting solution was transferred to a 25 mL Teflon-lined autoclave and subjected to a hydrothermal treatment at 180 °C for 4 h, producing a clear light green/blue solution without observable copper precipitation. The solution was then transferred to a crucible, mixed with 0.8 g of urea, and pyrolyzed at 500 °C with a heating rate of 10 °C min^−1^ in a tube furnace under an argon atmosphere.

Depending on the initial amount of citric acid (500, 250, or 150 mg), the resulting black powders were denoted as L1 (5 wt%), L2 (20 wt%), L3 (40 wt%), and L4 (60 wt%) Cu-SACs, respectively. The reported copper loadings correspond to nominal values calculated based on the precursor ratios, as defined in eqn (S1) in the SI.

### Material characterization

X-ray diffraction (XRD) patterns were obtained using a Bruker AXS D8 Advance X-ray diffractometer equipped with Cu Kα radiation source (*λ* = 1.54 Å) and a LYNXEYE detector. Thermogravimetric analysis (TGA) was performed to assess the weight percentage of metal present in SAC samples using a TA Instruments TGA 5500. Measurements were conducted in air by elevating temperature from 30 °C to 750 °C at a heating rate of 20 °C min^−1^. The Brunauer–Emmett–Teller (BET) surface area and pore size distribution of catalysts were determined by N_2_ adsorption–desorption isotherm measurements using a 3Flex instrument (Micromeritics). The surface area and pore size distributions were calculated using the Harkins–Jura *t*-plot method for the meso- and macropore regions.

Electrolyte samples were analyzed using inductively coupled plasma optical emission spectroscopy (ICP-OES; Agilent 5110 SVDV) in axial mode after 10× dilution in 1% v/v hydrochloric acid.

X-ray photoelectron spectroscopy (XPS) measurements were carried out using a Thermo Scientific K-Alpha photoelectron spectrometer with monochromatic Al Kα radiation (*hν* = 1486.6 eV). The X-ray beam was focused to a spot size of 400 µm. A flood gun was employed to reduce surface charging during analysis.

Aberration-corrected, high-angle annular dark-field scanning transmission electron microscopy (HAADF-STEM) imaging was performed using a Nion UltraSTEM 100 operated at 100 kV. For STEM characterization, the samples were mounted on lacey-carbon TEM grids (300-mesh copper, Ted Pella). Energy-dispersive X-ray spectroscopy (EDS) analysis was conducted using a JEOL NEOARM aberration-corrected TEM/STEM system.

### Electrochemical characterization

For ORR activity measurements, catalyst inks were prepared by dispersing 1 mg of Cu-SAC, 1 mg of carbon black, and 20 µL of Nafion solution in 1 mL of deionized water, followed by ultrasonication for 1 h. An aliquot (30 µL) of the ink was drop-cast onto a polished glassy carbon rotating disk electrode (RDE, 3 mm diameter) and dried under ambient conditions.

Electrochemical measurements were carried out using a CHI 760D potentiostat in a single-compartment cell containing 0.1 M KOH, employing a standard three-electrode configuration with Ag/AgCl as the reference electrode and a Pt wire as the counter electrode. Cyclic voltammetry (CV) was performed between 1.3 and 0.25 V (*vs.* RHE) at a scan rate of 50 mV s^−1^ in N_2_-saturated electrolyte. ORR activity was evaluated by linear sweep voltammetry (LSV) under O_2_-saturated conditions at rotation rates from 400 to 1600 rpm, with continuous O_2_ purging. The ORR performances of L1, L2, L3, and L4 Cu-SACs were compared with commercial 20 wt% Pt/C under identical conditions.

Catalyst durability was assessed using two complementary methods. First, the RDE-supported catalyst was subjected to 250 CV cycles between 1.3 and 0.25 V (*vs.* RHE) at 50 mV s^−1^ and 0 rpm, followed by LSV measurements at 1600 rpm to evaluate activity decay. Second, a catalyst ink containing 2 mg of SAC and Nafion was drop-cast onto a glassy carbon plate and tested in a bottom-neck electrochemical cell (Fig. S7) to examine post-cycling structural changes.

The electrical resistance (*R*_Ω_) from electrolyte and catalyst was determined from electrochemical impedance spectroscopy (EIS). EIS measurements were carried out with an amplitude of 10 mV at 0.4 V *vs.* RHE over a frequency range from 1 MHz to 1 Hz.

## Results and discussion

XRD patterns of L1, L2, L3, and L4 catalysts are shown in Fig. S1a. All samples exhibit a broad diffraction feature centered at ∼26.8°, characteristic of amorphous carbon formed by the carbonization of carbon nanodots during pyrolysis. At lower metal precursor loadings (L1 and L2), no diffraction peaks corresponding to crystalline copper are detected, indicating that copper is predominantly present in an atomically dispersed or highly disordered state below the XRD detection limit. In contrast, at higher loadings (L3 and L4), distinct diffraction peaks associated with metallic Cu emerge, reflecting the onset of copper aggregation and nanoparticle formation. This transition highlights the strong dependence of atomic dispersions on metal precursor loading, where increasing copper content exceeds the available anchoring capacity of carbon–nitrogen framework, leading to metal–metal interaction and metal nanoparticle formation.

To assess relative copper content in these materials, thermogravimetric analysis (TGA) was performed in air (Fig. S1b). The residual mass after carbon combustion increases systematically with nominal copper loading (Table S1), indicating progressive incorporation of copper into the carbon framework. When considered together with the XRD results, these data show that increasing precursor loading effectively raises the total copper content, however atomic dispersion is preserved only up to an optimal loading (L2), beyond which excess copper aggregates into nanoparticles. For accurate quantification of copper content, ICP-OES analysis was conducted for the best-performing catalyst, L2, which revealed a copper loading of 19.2 wt%. Previously reported Cu single-atom catalysts commonly exhibit low metal loadings, typically below 1 wt% while maintaining atomic dispersion.^23^ Although some studies have reported higher metal loadings of 1–5 wt% range,^24^ achieving stable and well-dispersed single-atom configurations at such loadings remains challenging. In this context, the achievement of ∼20 wt% Cu loading in the present work represents a substantial advancement and can be considered an ultrahigh-loading single-atom catalyst.^25^

**Fig. 2:**
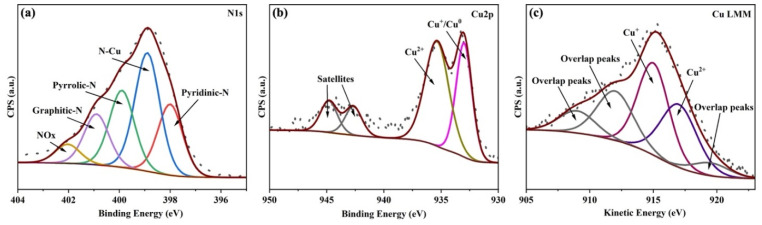
High-resolution XPS (a) N 1s and (b) Cu 2p spectra for L2 and (c) Cu LMM Auger spectrum for the same catalyst.

X-ray photoelectron spectroscopy (XPS) was employed to probe the elemental composition and chemical states of Cu and N in the Cu-SACs. XPS survey spectra of L2, L3, and L4 are shown in Fig. S2, and the corresponding atomic percentages of C, N, O and Cu are summarized in Table S2. High-resolution N 1s and Cu 2p spectra of the optimally loaded L2 catalyst are presented in Fig. 2, while those of the higher-loading samples (L3 and L4) are shown in Fig. S3. Notably, the carbon-dot-derived synthesis yields ultrahigh nitrogen contents (23–27 at%) across L2, L3, and L4, substantially exceeding values typically reported for M–N–C catalysts prepared by conventional methods.^26,27^ This elevated nitrogen content provides a high density of coordination sites, which is critical for capturing and stabilizing atomically dispersed copper during the pyrolysis-driven transformation of carbon nanodots to conductive carbon framework.

The high-resolution N 1s XPS spectrum of L2 (Fig. 2a) was deconvoluted into four basis peaks centered at 398.3, 399.1, 400.1, and 401.1 eV, which are assigned to pyridinic-N, Cu–N, pyrrolic N, and graphitic N chemical states, respectively.^28,29^ Notable, the Cu–N component accounts for 32.3% of total nitrogen content (Table S3), indicating that a substantial fraction of nitrogen sites participate in Cu coordination. This high density of Cu–N coordination environments is consistent with the stabilization of atomically dispersed copper at the optimal loading.

The high-resolution Cu 2p XPS spectrum of L2 (Fig. 2b) showed two characteristic features centered at 933.0 and 935.3 eV, which are assigned to Cu^0^/Cu^+^ and Cu^2+^, respectively.^28,29^ In contrast, for the higher-loading samples L3 and L4 (Fig. S3), the Cu^0^/Cu^+^ feature shifts to lower binding energy (∼932.3 eV) with enhanced intensity, indicating the emergence of metallic copper species, in agreement with the XRD results. Because Cu 2p XPS spectra alone cannot distinguish Cu^+^ from Cu^0^, Cu LMM Auger spectroscopy was employed for definitive oxidation-state assignment (Fig. 2c for L2 and Fig. S4 for L3 and L4). Deconvolution of the Auger spectra reveal distinct contributions at approximately 915 eV (Cu^+^), 917 eV (Cu^2+^), and 918 eV (Cu^0^), along with overlapping multiplet features at 908.8, 911.9, and 919.6 eV.^30–34^ Quantitative analysis of the combined Cu 2p XPS and Cu LMM Auger results (Table S4) shows a clear loading-dependent evolution of copper species: Cu^0^ content is negligible in L2 but increases to 14% in L4, while the Cu^2+^ fraction gradually decreases from 40% (L2) to 25% (L4). Several previous studies reported that Cu–N_2_ coordination plays a critical role in promoting efficient ORR by providing undercoordinated Cu active sites for adsorption of O_2_.^35,36^ Importantly, at the optimal loading (L2), atomically dispersed copper is mainly present as Cu^+^ species, with the smaller fraction of Cu^2+^, consistent with Cu–N coordination environments.

The surface area and pore size distribution of the L2 catalyst were determined by N_2_ gas adsorption–desorption isotherm measurements (Fig. S5). L2 exhibits a relatively low BET surface area of 19.4 m^2^ g^−1^, with a pore size distribution dominated by mesopores, primarily larger than 10 nm, resulting in a cumulative mesopore surface area of 11.5 m^2^ g^−1^. The limited surface area and prevalence of large mesopores suggest that a fraction of Cu–N active sites may be partially embedded within the carbon matrix, thereby reducing their electrochemical accessibility. Future optimization of pore size and pore distribution, for example through the introduction of sacrificial templates during synthesis, may further enhance active-site exposure and catalytic utilization.

**Fig. 3:**
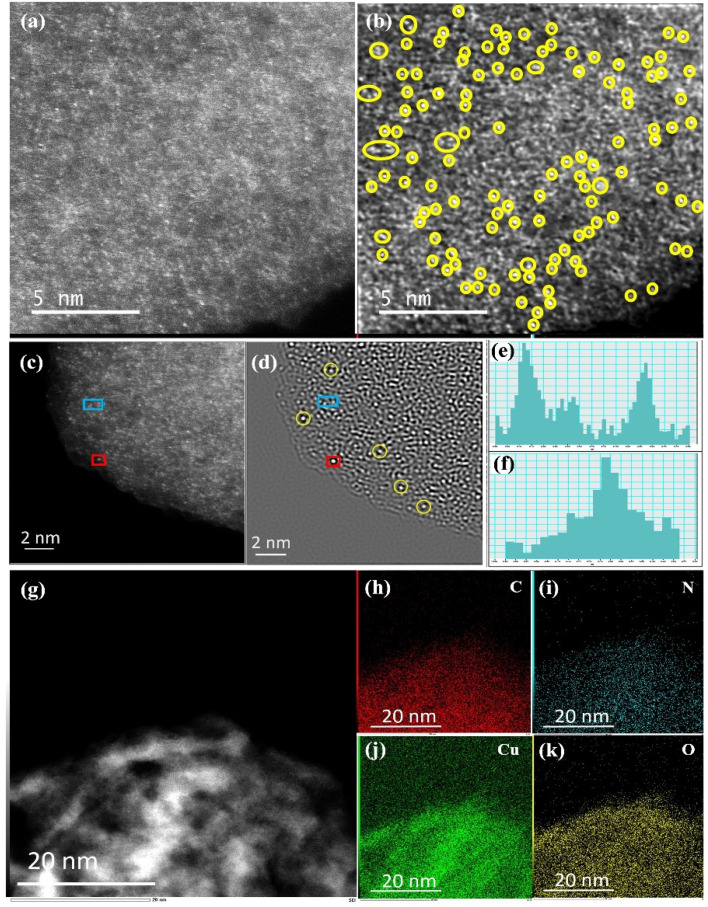
(a) Atomic scale HAADF-STEM image of the L2 catalysts showing Cu single atoms as bright spots in the C matrix, and (b) the corresponding IFFT filtered image (from (a)) highlighting Cu atoms and clusters (marked with yellow circles). (c) HAADF-STEM image of the same catalyst in another location and (d) the corresponding IFFT-filtered image highlighting Cu atoms (marked with yellow circles). (e and f) Line-scan intensity profiles across individual Cu single atoms as indicated by the blue and red rectangles in (c and d), demonstrating localized Cu signal variation relative to the surrounding carbon support. (g–k) EDS mapping of C, N, Cu, and O in the L2 catalyst.

Aberration-corrected HAADF-STEM was employed to directly visualize the dispersion of copper in the L2 catalyst. As shown in Fig. 3a and b, numerous isolated bright spots are observed against the carbon background and are attributed to individual Cu atoms due to their higher atomic number relative to C, N, and O. The measured size of these features (∼0.13 nm) is consistent with the atomic radius of Cu, confirming atomic-scale dispersion within the pyrolyzed carbon framework. While copper is predominantly present as isolated single atoms, a small number of disordered atomic clusters are also observed in localized regions (Fig. 3b). The absence of lattice fringes indicates that these clusters are noncrystalline and limited in extent, consistent with XRD and XPS results showing atomic dispersion as the dominant copper species in L2.

HAADF-STEM (Fig. 3c) and IFFT-filtered image (Fig. 3d) obtained in another location of the same catalyst, L2, enable the visualization of Cu atomic features within the carbon matrix. Fig. 3e and f show the line-scan profiles collected across a copper-grouped region and copper-isolated region, respectively. Direct visualization of nitrogen coordination at the atomic scale is challenging due to the similar atomic numbers of N and C, which limits Z-contrast in STEM. Nevertheless, the predominance of isolated Cu intensity profiles, together with the high Cu–N fraction identified by XPS, provides complementary evidence supporting Cu–N-coordinated, atomically dispersed copper as the dominant species in the L2 catalyst.

The EDS spectrum (Fig. S6) of the L2 catalyst confirms the presence of C, N, O, and Cu, in agreement with the XPS results. The dominant Cu signal in the EDS spectrum is primarily attributed to the copper mesh of the TEM grid. Elemental mapping by energy-dispersive X-ray spectroscopy (EDS) (Fig. 3g–k) reveals a homogeneous spatial distribution of Cu and N throughout the carbon matrix, indicating effective incorporation of copper into nitrogen-rich coordination environments during pyrolysis. Oxygen is also uniformly distributed and present in a substantial amount, originating from oxygen-containing functional groups of citric-acid-derived carbon nanodots. While such oxygen functionalities may assist in metal anchoring, their high concentration could adversely affect electrical conductivity, suggesting that further optimization of heteroatom composition and precursor chemistry may be beneficial for improving charge transport in future designs.

**Fig. 4:**
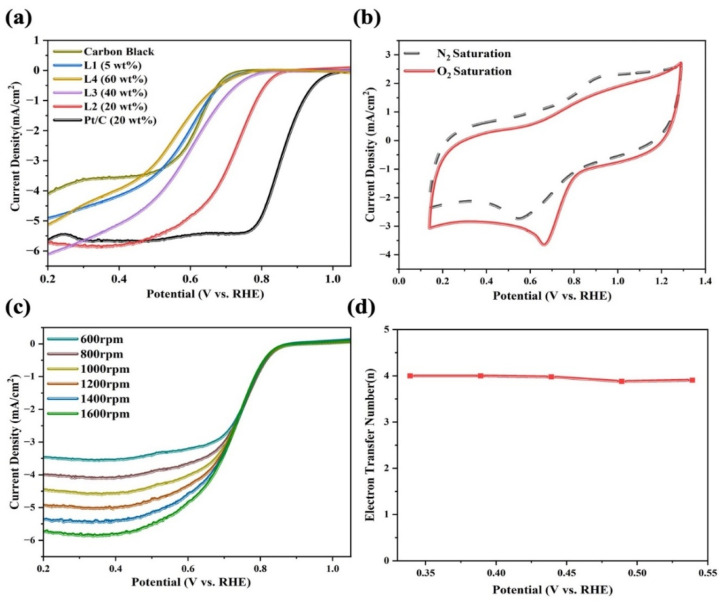
ORR catalytic activity of Cu-SACs: (a) LSVs (1600 rpm) of L1, L2, L3, L4 Cu-SACs along with carbon black and Pt/C in O_2_ saturated 0.1 M KOH, (b) CVs recorded for L2 in N_2_- and O_2_-saturated 0.1 M KOH, (c) LSV curves of L2 at different rotation rates (600–1600 rpm), and (d) electron transfer number (*n*) of L2 determined by K–L analysis.

The ORR activity of the Cu-SAC catalysts was evaluated using RDE in O_2_-saturated 0.1 M KOH, with commercial 20 wt% Pt/C used as a benchmark. Fig. 4a shows LSV curves recorded at 1600 rpm (10 mV s^−1^). The onset potentials, defined at a current density of −0.1 mA cm^−2^, were 0.85, 0.79, and 0.75 V for L2, L3, and L4, respectively, compared with 1.0 V for Pt/C. Among the Cu-SACs, L2 exhibited the most positive onset potential, which is 0.15 V lower than that of Pt/C. The high limiting current of L2 is consistent with efficient ORR kinetics and suggests a dominant four-electron pathway, which is subsequently confirmed by Koutecky–Levich analysis. The superior ORR performance of L2 is attributed to the high abundance of Cu^+^–N_2_ coordination, which provides under-coordinated active sites with a lower activation energy for ORR compared to Cu^2+^–N_4_ configurations.^28,29^ In contrast, L3 and L4, despite having comparable Cu^+^ contents, exhibit lower ORR activity, which is primarily attributed to the formation of copper nanoparticles at higher loadings that block or encapsulate Cu^+^–N_2_ active sites, thereby reducing electrochemically accessible site density.

Fig. 4b shows cyclic voltammograms (CVs) of the L2 catalyst recorded under N_2_- and O_2_-saturated conditions. Under an N_2_ atmosphere, the CV displays distinct redox peaks at approximately 0.55 and 0.95 V, which are attributed to reversible copper redox transitions. In contrast, under O_2_-saturated conditions, a pronounced cathodic peak appears at ∼0.66 V, confirming the oxygen reduction activity of L2. Although L2 exhibits superior ORR performance among the Cu-SACs, BET analysis indicates a relatively low surface area, suggesting that a fraction of Cu^+^–N_2_ active sites may remain embedded within the carbon matrix and are not fully electrochemically accessible.^37^

We have compared the performance of L2 with previously reported Cu-SAC systems featuring high surface areas (typically 500–1600 m^2^ g^−1^) and well-developed pores structures. As shown in Table S7, L2 exhibits are relatively lower half-wave potential than highly porous catalysts, resulting from the limited mass transport and accessibility of active sites. Consequently, regulating the pore size distribution and increasing the accessibility of Cu^+^–N_2_ sites in L2 could further enhance ORR performance by exposing a greater number of active sites to the electrolyte.

To further elucidate the ORR kinetics and electron transfer pathway of the L2 catalyst, LSV measurements were conducted at different rotation rates ranging from 400 to 1600 rpm (Fig. 4c). The increase in current density with increasing rotation rate reflects enhanced convective mass transport induced by electrode rotation, which reduces the diffusion boundary layer thickness and facilitates oxygen delivery to the catalyst surface. The corresponding Koutecky–Levich plots (Fig. S7) exhibit good linearity between *j*^−1^*vs. ω*^−1/2^. The electron transfer numbers (*n*) determined from the slopes of the Koutecky–Levich plots are close to four over the investigated potential range, demonstrating that the ORR on the L2 catalyst predominantly proceeds *via* a four-electron pathway (Fig. 4d). This result is consistent with the high limiting current density observed in Fig. 4a and further supports the effective role of Cu^+^–N_2_ active sites in promoting direct oxygen reduction to OH^−^ in alkaline media.

The electrical conductivity of the L2 catalyst was evaluated by EIS. The ohmic resistance of L2 (∼70 Ω) is comparable to that of carbon black, indicating that the inclusion of carbon black effectively mitigates intrinsic conductivity limitation of the SAC.

**Fig. 5:**
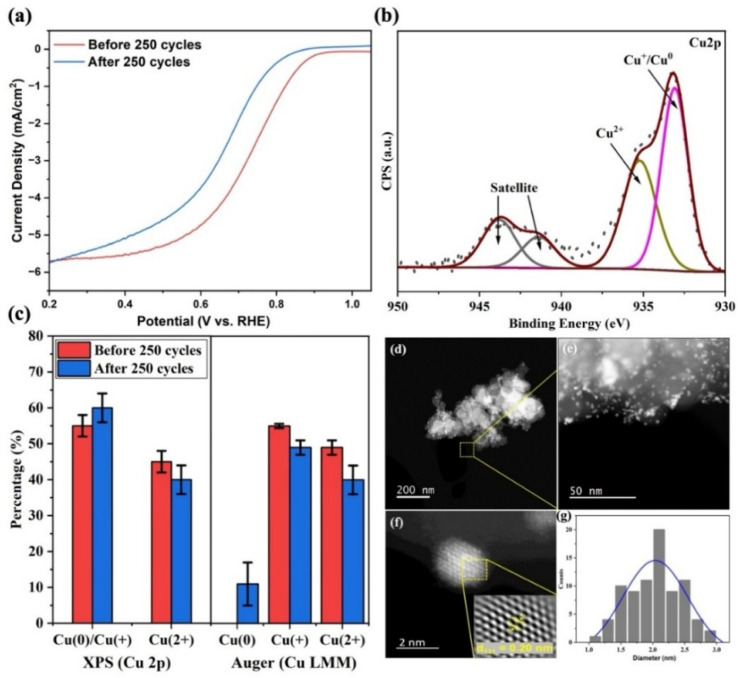
Stability of L2 catalyst after 250 cycles: (a) LSV curves of fresh and cycled L2 recorded with RDE at 1600 rpm, (b) high-resolution Cu 2p XPS spectrum of post-cycled L2, (c) comparison of copper chemical states before and after cycling by XPS and Auger analysis, (d and e) HAADF-STEM images of L2 after cycling at low magnification, (f) high-resolution STEM image of a Cu nanoparticle with the inset showing an IFFT-filtered lattice image (*d*-spacing = 0.20 nm); and (g) histogram of Cu nanoparticle size distribution.

The durability of the L2 (20 wt%) Cu-SAC catalyst was evaluated by continuous CV cycling between 1.3 and 0.25 V (*vs.* RHE) for 250 cycles in O_2_-saturated 0.1 M KOH. The catalyst ink of L2 was drop-cast onto a glassy carbon plate and tested in a bottom-neck electrochemical cell (Fig. S8) to examine post-cycling structural changes. Fig. 5a compares the linear sweep voltammetry (LSV) curves recorded at 1600 rpm before and after cycling. A noticeable negative shift in onset potential from 0.89 to 0.82 V (at 0.2 mA cm^−2^) is observed after 250 cycles, accompanied by a decrease in current density, indicating degradation of ORR activity under prolonged electrochemical operation. Also, we compared the durability of L2 with previously reported high-loading Cu-SAC (Table S6). As shown in the table, the reported durability of Cu-based SACs varies, with activity losses ranging from 7% to 25% under potentiostatic conditions. Direct comparison with our results is not straightforward, as our stability test was conducted under 250 continuous CV cycling (corresponding to approximately 3 h of testing), our catalyst exhibits more pronounced degradation. Overall, this comparison indicates that, while the Cu-SAC synthesized in this study demonstrates advantages such as high metal loading (up to ∼ 20 wt%) and elevated nitrogen content (23–27 at%), there remains significant room to further enhance stability. In particular, improving the robustness of Cu active sites will require optimization of the M–N–C coordination environment and the carbon framework.

To elucidate the origin of this performance decay, post-cycling catalysts were examined using XPS, STEM, and ICP analyses. As shown in Fig. 5b, the high-resolution Cu 2p spectrum of the L2 catalyst after 250 cycles exhibits a slight shift of the Cu^0^/Cu^+^ peak toward lower binding energy (932.3 eV) compared to the pristine catalyst (933.0 eV), together with a reduction in the relative intensity of Cu^+^ and Cu^2+^ species. Consistent trends are observed in the Cu LMM Auger spectra (Fig. S9), indicating progressive reduction and restructuring of copper species during ORR cycling. Quantitative analysis (Fig. 5c and Table S5) confirms a substantial increase in metallic Cu^0^ (∼10%) at the expense of Cu^+^–N and Cu^2+^ species, suggesting demetallation and loss of metal–nitrogen coordination under electrochemical conditions.

Direct structural evidence for catalyst restructuring is provided by HAADF-STEM imaging of the cycled L2 catalyst (Fig. 5d–f). In contrast to the atomically dispersed copper observed in the pristine sample, numerous copper nanoparticles are formed after 250 cycles. High-resolution images reveal well-defined lattice fringes with an interplanar spacing of 0.20 nm, corresponding to the Cu(111) plane, confirming the metallic nature of the nanoparticles. Statistical analysis of 81 particles (Fig. 5g) shows a narrow size distribution with an average diameter of 2.0 ± 0.5 nm.

Additionally, ICP-OES analysis of the electrolyte was conducted after 250 continuous CV cycles (corresponding to approximately 3 h of testing). The results revealed that 6.7% (±1.0%) of Cu was leached into the electrolyte during electrolysis.

The XPS, STEM, and ICP-OES analyses after electrolysis confirm that both Cu dissolution and aggregation into Cu^0^ contribute to the observed degradation of ORR activity in L2. The loss of metal–nitrogen coordination likely initiates Cu dissolution and nanoparticle formation, leading to a decrease in active site density and catalytic performance.^38–41^ These results highlight the intrinsic stability challenge of high-loading Cu single-atom catalysts under ORR conditions and underscore the importance of simultaneously stabilizing metal coordination environments and optimizing carbon supports to suppress demetallation and aggregation.

Overall, these results suggest that, beyond achieving high metal loading, optimizing pore structure and surface area is essential to fully realize the catalytic potential of single-atom catalysts. To construct highly porous, well-structured catalysts while maintaining high Cu loading, future strategies may include incorporating removable salt templates^42^ during pyrolysis or using SiO_2_ sacrificial templates^43^ to generate hierarchical pore structures. These approaches could enhance O_2_ mass transport and improve the accessibility of Cu^+^–N_2_ active sites without compromising the high-loading capability of the CND-derived synthesis.

## Conclusion

In conclusion, we present a simple and scalable strategy to synthesize copper single-atom catalysts (Cu-SACs) using citric-acid-derived carbon nanodots as metal-capturing precursors, enabling ultrahigh copper loadings with atomic dispersion. Among the catalysts studied, the L2 sample (20 wt% Cu) exhibits the most favorable ORR performance, delivering a limiting current density comparable to that of commercial 20 wt% Pt/C and predominantly following a four-electron pathway in alkaline media. These results demonstrate that high intrinsic ORR activity can be achieved in non-precious metal SACs through effective control of metal–nitrogen coordination.

Durability tests, however, reveal that the L2 catalyst suffers from activity decay during prolonged electrochemical operation. Post-cycling analyses indicate demetallation and aggregation of copper single atoms into metallic nanoparticles, leading to a loss of Cu^+^–N_2_ active sites. This highlights an inherent trade-off between activity and stability in high-loading Cu-SACs. Overall, this work establishes carbon nanodots as a promising platform for high-loading SAC synthesis while identifying key challenges related to active-site accessibility and long-term stability that must be addressed for practical ORR applications.

## Conflicts of interest

There are no conflicts of interest to declare.

## Supplementary Material

RA-OLF-D6RA01912A-s001

## Data Availability

The data underlying this study are available in the published article and its supplementary information (SI). Supplementary information: the results of XRD, TGA, XPS, Auger spectroscopy, and Koutecky–Levich analyses. See DOI: https://doi.org/10.1039/d6ra01912a.
