# Supplementary material for: Carbon nanodot precursors enable ultrahigh-loading copper single-atom catalysts for oxygen reduction reaction
Source: RSC Adv. 2026 Jul 6. Online ahead of print. doi: 10.1039/d6ra01912a (PMC13334702; doi:10.1039/d6ra01912a)
Supplement: RA-OLF-D6RA01912A-s001 [file RA-OLF-D6RA01912A-s001.pdf]

Supplementary Information (SI) for RSC Advances.
This journal is © The Royal Society of Chemistry 2026

Supporting Information

Carbon Nanodot Precursors Enable Ultrahigh-Loading Copper Single-Atom Catalysts for Oxygen Reduction Reaction

Prakhar Sharma[a], Ayanthi Thisera[a], Matthew G. Boebinger[b], Jenna Rector[a], Jason M. Unrine[c], Beth S. Guiton[a], Doo Young Kim[a,*]

[a]Department of Chemistry, University of Kentucky, Lexington, Kentucky 40506, United States

[b]Center for Nanophase Materials Sciences, Oak Ridge National Laboratory, Oak Ridge, Tennessee 37831, United States

[c]Department of Plant and Soil Sciences, University of Kentucky, Lexington, Kentucky 40506, United States

Corresponding Author

Doo Young Kim – Email: dooyoung.kim@uky.edu

Equation S1.

$$Nominal\ wt\% = \frac{Mass_{added\ Cu}(mg)}{Mass_{added\ Cu}(mg) + Mass_{CNDs\ from\ CA\ used}(mg)} \times 100\%$$

Table S1. TGA of 5, 20, 40 and 60wt% of Cu SAC

Cu SAC sample	Cu nominal wt%	Residue mass in TGA (wt%)
L1	5	4.9
L2	20	21.8
L3	40	35.1
L4	60	63.5

Table S2. Atomic percentage of nitrogen, carbon, oxygen, and copper in L2, L3, and L4 Cu-SACs.

sample	C (at. %)	N (at. %)	O (at. %)	Cu (at. %)
L2	62 ± 6	27.0 ± 0.8	8.6 ± 0.6	2.4 ± 0.2
L3	56.9 ± 2.6	25.6 ± 1.5	12 ± 1	5.6 ± 2.6
L4	56.0 ± 2.3	23.2 ± 0.8	12.7 ± 0.6	7.6 ± 2.2

Table S3. Fraction of nitrogen chemical states in L2

Pyridinic N (at.%)	Cu-N (at.%)	Pyrrolic N (at.%)	Graphitic N (at.%)
20.5 ± 2.3	32.3 ± 4.6	23.8 ± 1.1	16.3 ± 1.9

Table S4. Copper chemical states determined by XPS and Auger spectroscopic analysis

catalyst	Total copper content (at. %)	XPS analysis		Auger spectroscopy		
		Cu^0/Cu^+ (%)	Cu^{2+} (%)	Cu^0 (%)	Cu^+ (%)	Cu^{2+}(%)
L2	2.4 ± 0.1	55 ± 3	45 ± 3	0	55 ± 6	45 ± 7
L3	5.6 ± 2.6	64.8 ± 2.4	35.2 ± 2.4	6.7 ± 1.2	58.2 ± 2.7	35.0 ± 2.2
L4	7.6± 2. 2	67.0 ± 4.7	32.9 ± 4.6	14.0 ± 1.1	56.9 ± 2.4	29.1 ± 2.2

Table S5. Change of **c**opper chemical states in L2 after cycling.

	XPS		Auger spectroscopy		
	Cu^0/Cu^+ (%)	Cu^{2+} (%)	Cu^0 (%)	Cu^+ (%)	Cu^{2+} (%)
Initial	55 ± 3	45 ± 3	0	55 ± 6	45 ± 7
After 250 cycles	60 ± 4	40 ± 4	11 ± 6	49 ± 2	40 ± 4

Table S6. Comparison of stability between L2 and previously reported high-loading Cu single atom catalysts

SACs	Copper loading (wt%)	Current decay	Reference
L2	19.2	30 % decrease at 0.65 V after 250 CV cycles (approximately 3 h)	This work
Cu-N-C	5.4	7 % decrease at 0.65 V after 10 h chronoamperometry	[1]
Cu-N-C	10.9	25 % decrease at 0.7 V for 10 h chronoamperometry	[2]
Fluorine-modified Cu-N-C	9.8	17 % decrease at 0.7 V for 10 h chronoamperometry	[3]

Table S7. Comparison with previously reported Cu-SACs with high surface area.

Cu-SACs on different carbon	BET Surface Area (m^2/g)	Cu loading (wt%)	Half-wave potential (V vs. RHE) in 0.1 M KOH	Reference
L2	19.2	19.2	0.72	This work
3d nitrogen doped porous carbon	529.5	0.22	0.74	[41
Nitrogen doped carbon derived from ZIF-8	632	2.1	0.85	[5]
Porous carbon derived from melamine	361	1.3	0.88	[6]
Microporous carbon spheres.	1450	1.1	0.90	[7]

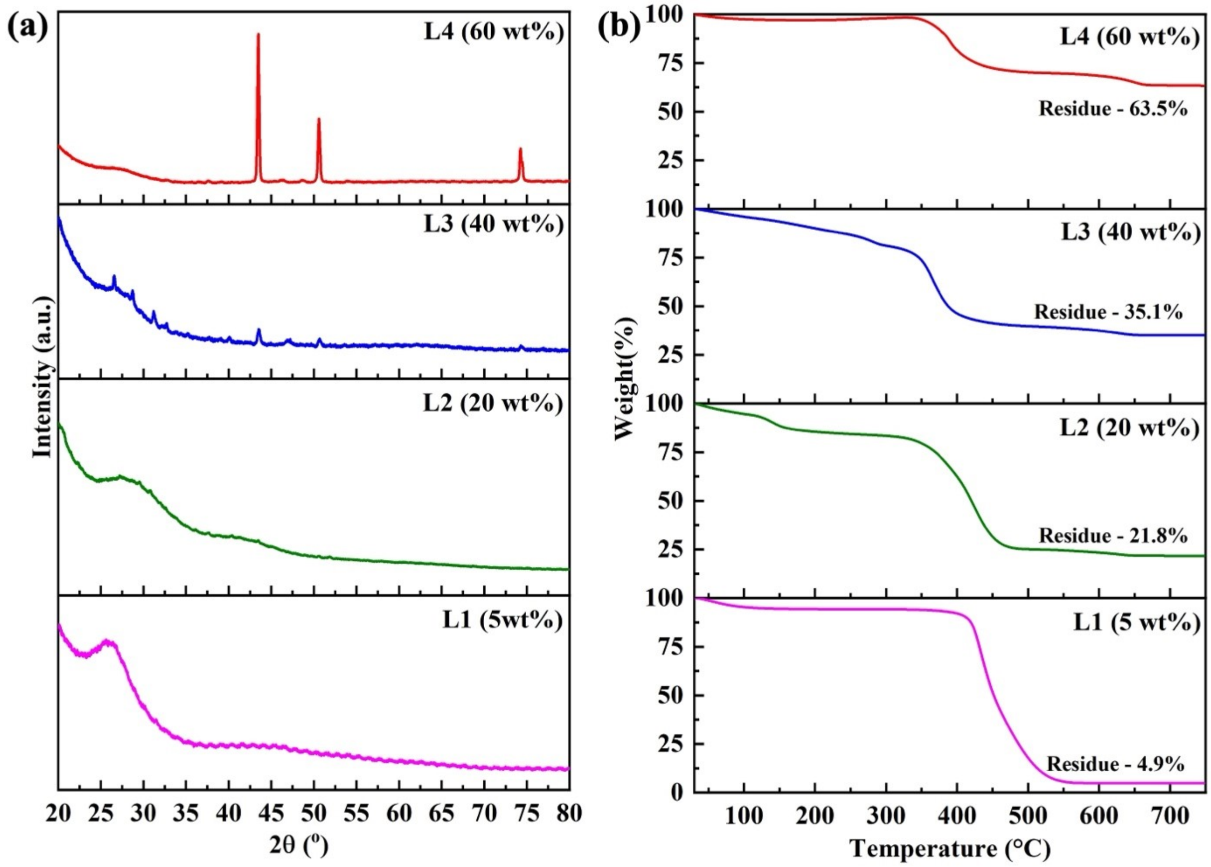

Figure S1. (a) XRD patterns and (b) thermogravimetric analysis of L1, L2, L3 and L4

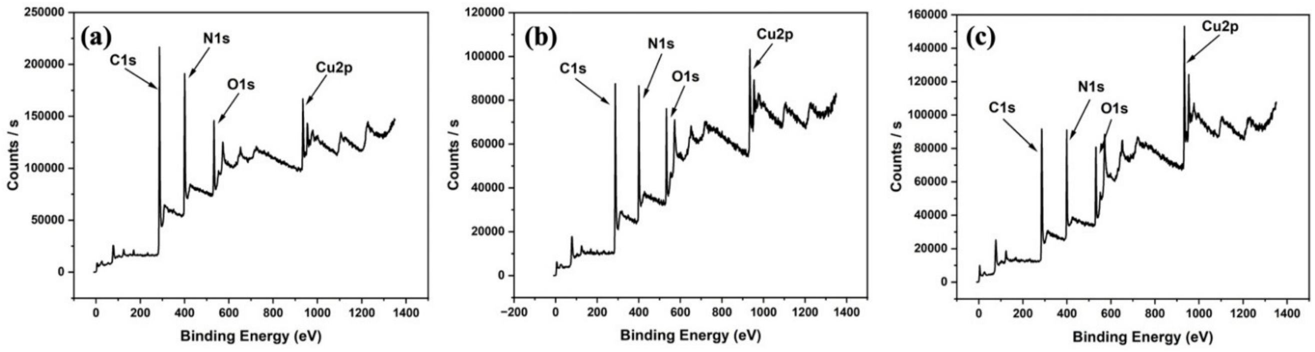

Figure S2. XPS survey spectra of (a) L2, (b) L3, and (c) L4.

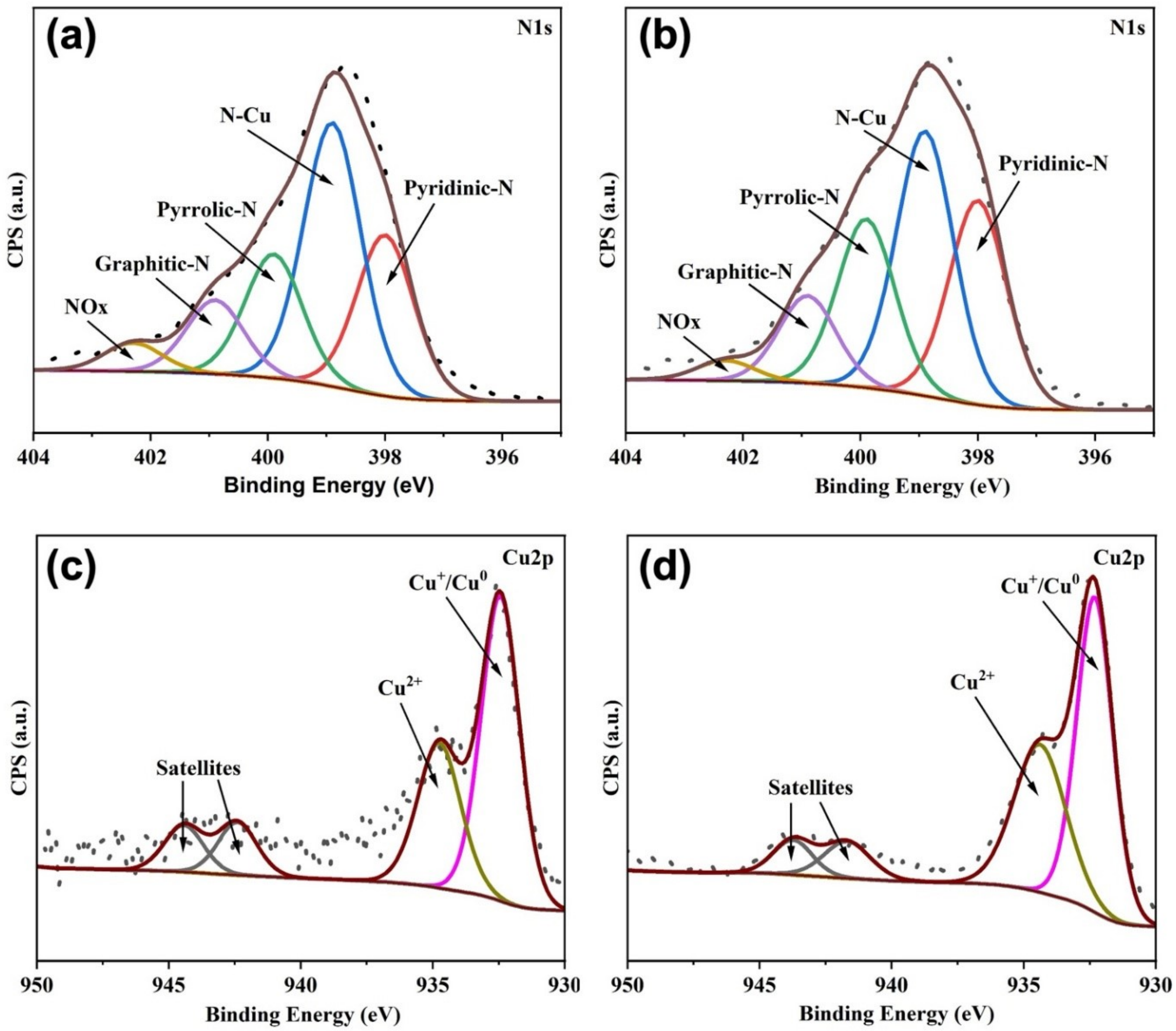

Figure S3. High resolution XPS N1s spectra and deconvoluted peaks for (a) L3 and (b) L4; high resolution Cu 2p spectra and deconvoluted peaks for (c) L3 and (d) L4

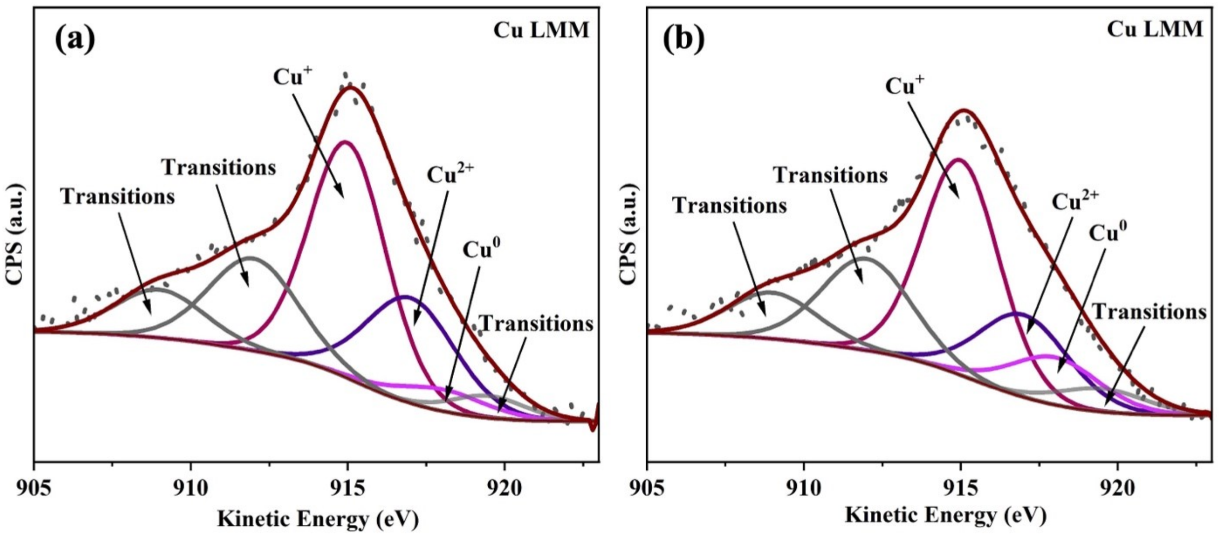

Figure S4. Cu LMM Auger spectra of (a) L3 and (b) L4

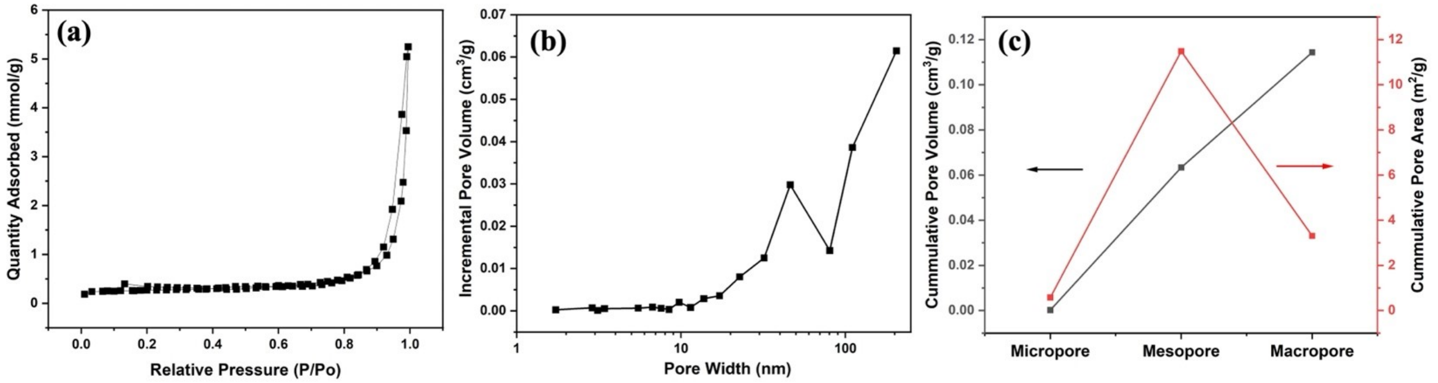

Figure S5. BET surface area and pore distribution of the L2 catalyst: (a) isothermal N_2 adsorption/desorption plot, (b) pore size distribution, and (c) cumulative pore volume and pore areas of micropore, mesopore and macropore.

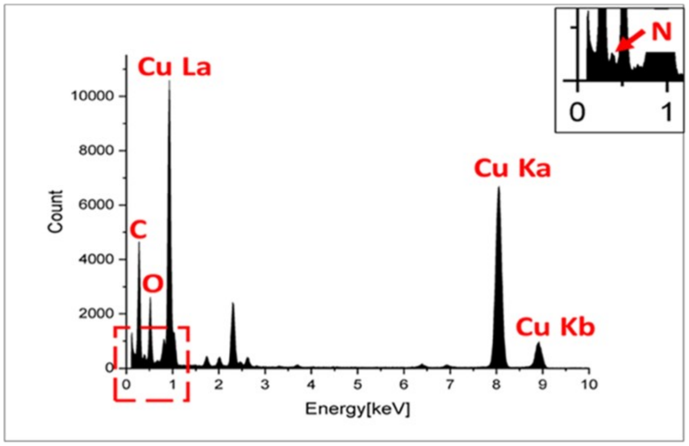

Figure S6. EDS spectrum of the L2 catalyst, confirming the presence of C, N, O, and Cu.

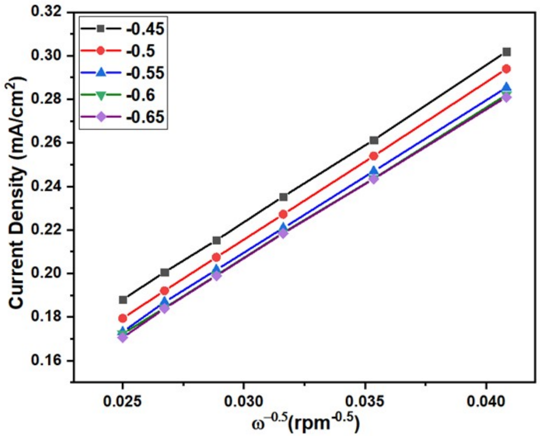

Figure S7: Koutecky-Levich plots of the L2 catalyst.

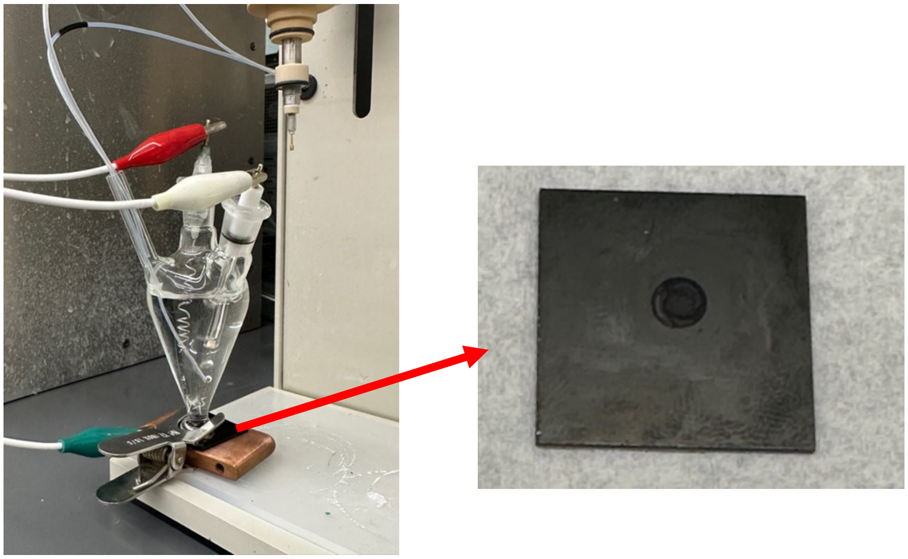

Figure S8: experimental setup for electrochemical stability testing.

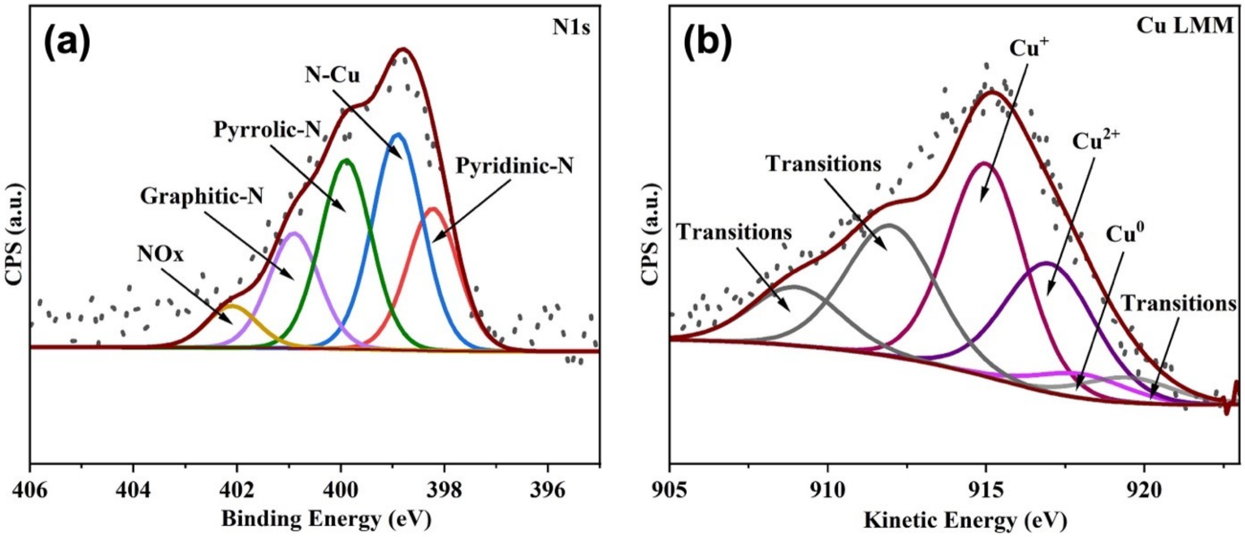

Figure S9: (a) N1s XPS spectrum and (b) Auger spectrum of the L2 catalyst after 250 cycles.

References

1. G. Han et all, Nano energy, (2019), 66, 104088
2. ACS Appl. Energy Mater. (2025), 8, 12, 8207–8215
3. J. Yang et al., ACS Appl. Energy. Mater. (2025), 6, 12, 8217
4. M. Zhang, H. Li, J. Chen, F. -X. Ma, L. Zhen, Z. Wen, C.-Y. Xu, *Small* 2022, 18, 2202476
5. Anand Parkash *J. Electrochem. Soc.* 2020, **167** 155504
6. L. Wu, Y. Wang, C. Shao, L. Wang, B. Li*, J. Mater. Chem. A*, 2025,13, 5974-5986
7. L.Zong, K.Fan, W.Wu, L.Cui, L.Zhang, B.Johannessen, D.Qi, H.Yin, Y.Wang, P.Liu, L.Wang, H .Zhao, *Adv. Func. Mater.* 2021, 31, 2104864.
